# CausNet-partial: ‘Partial Generational Orderings’ based search for optimal sparse Bayesian networks via dynamic programming with parent set constraints

**DOI:** 10.1371/journal.pone.0324622

**Published:** 2025-06-10

**Authors:** Nand Sharma, Joshua Millstein

**Affiliations:** Division of Biostatistics, Department of Population and Public Health Sciences, University of Southern California, Los Angeles, California, United States of America; University of Essex, UNITED KINGDOM OF GREAT BRITAIN AND NORTHERN IRELAND

## Abstract

In our recent work, we developed a novel dynamic programming algorithm to find optimal Bayesian networks with parent set constraints. This ‘generational orderings’ based dynamic programming algorithm—CausNet—efficiently searches the space of possible Bayesian networks. The method is designed for continuous as well as discrete data, and continuous, discrete and survival outcomes. In the present work, we develop a variant of CausNet—CausNet-partial—where we introduce the space of ‘partial generational orderings’, which is a novel way to search for small and sparse optimal Bayesian networks from large dimensional data. We test this method both on simulated and real data. In simulations, CausNet-partial shows superior performance when compared with three state-of-the-art algorithms. We apply it also to a benchmark discrete Bayesian network ALARM, a Bayesian network designed to provide an alarm message system for patient monitoring. We first apply the original CausNet and then CausNet-partial, varying the partial order from 5 to 2. CausNet-partial discovers small sparse networks with drastically reduced runtime as expected from theory. To further demonstrate the efficacy of CausNet-partial, we apply it to an Ovarian Cancer gene expression dataset with 513 genes and a survival outcome. Our algorithm is able to find optimal Bayesian networks with different number of nodes as we vary the partial order. On a personal computer with a 2.3 GHz Intel Core i9 processor with 16 GB RAM, each processing takes less than five minutes. Our ‘partial generational orderings’ based method CausNet-partial is an efficient and scalable method for finding optimal sparse and small Bayesian networks from high dimensional data.

## 1 Introduction

Optimal Bayesian Network (BN) Structure Discovery is a method to learn optimal Bayesian networks from data (see e.g. [1–6]). Finding a best BN is NP-hard [7, 8]—the number of possible structures for *n* variables being 𝒪(n!2(n2)) [9]. The super-exponential size of the search space makes BN Structure Discovery through exhaustive search impossible for high dimensional data.

Optimal BN Structure Discovery methods are broadly classified as score-based (exact and approximate), constraint-based, and hybrid methods [10]. In the present work, we focus our attention to the score-based exact methods. The first exact methods that searched the whole space were based on Dynamic Programming (DP)—these include DP methods by Koivisto & Sood [11, 12], Silander and Myllymäki [13] and Singh and Andrew [14]. But even with dynamic programming, that reduces the complexity to exponential, the search is feasible only for very small data sets—usually less than 30 variables [13, 14], and even that is achieved by severely restricting the number of parents for each node.

Later exact algorithms worked on pruning the search space to find an optimal BN. The main methods in this category are GOBNILP (Globally Optimal Bayesian Network learning using Integer Linear Programming) [15] by Cussens that uses integer linear programming [15, 16] for pruning the search space; the A* search method by Yuan and Malone [17, 18]; and Branch-and-Bound methods (e.g. by de Campos *et al*. [19]). In [20], Fan *et al*. also implemented potentially optimal parent sets (POPS) and ancestral constraints to further prune the search space in the A* suite of algorithms. These exact methods improved upon the DP algorithms in terms of efficiency and speed and can be applied to higher number of variables [10].

On the similar lines, to overcome the curse of dimensionality in large datasets, we recently developed a dynamic programming based method CausNet [21] that identifies and implements ‘parent set constraints’ to prune the search space to find an optimal BN. In this method, we introduced a novel approach of ‘Generational orderings’ based search rather than the current lexicographic search for learning optimal BNs using dynamic programming.

In the current work, we introduce an extension of the CausNet approach where we consider ‘Partial Generational orderings’ rather than complete ‘Generational orderings’ to provide another novel way to tackle the curse of dimensionality in learning optimal BNs. This new method- CausNet-partial- further optimizes the search for small and sparse BNs in the subspace of ‘Partial Generational orderings’. This reduces the search space drastically and makes CausNet-partial work for large-dimensional data. The amount of reduction in search space can be controlled by the user according to the problem and prior domain knowledge, using specifiable parameters.

Many available algorithms do not support both continuous and discrete data [22], and few support survival outcomes [23]. Among the score-based exact methods, none of the algorithms by Koivisto & Sood [11], Silander and Myllymäki [13], Singh and Andrew [14] or GOBNILP [15] support survival outcomes. Even for other algorithms, little work has been done to support survival outcome in optimal BN learning [23]. To our knowledge, only a few methods like [24–26] support survival outcomes. CausNet and our new method CausNet-partial provide support for both continuous and discrete data (but not mixed data as of now), and also provide support for survival outcomes in the case of continuous data. As survival outcome is a scenario common in many biomedical data, this fills an important gap among the available BN learning methods.

## 2 Background

A Bayesian network [27, 28] is a probabilistic graphical model represented by a directed acyclic graph (DAG). The vertices $V=\{v_{1},\ldots ,v_{p}\}$ of the DAG correspond to random variables and the edges represent conditional dependences among the random variables. For each vertex $v_{i}$, a conditional probability distribution $P(v_{i}|parents(v_{i}))$ gives the dependence of the variable $v_{i}$ on its set of parents $parents(v_{i})$.

A Bayesian network *G* can be compactly represented by a vector $G=(G_{1},\ldots ,G_{p})$ : here *G*_*i*_ is a subset of *V* with directed edges to $v_{i}$. Any BN also corresponds to an ordering of the vertices, given by the ordered set $\left\{v_{\sigma_{i}}\},i\in \{1,2,\ldots ,p\right\}$, where σ is a permutation of [*p*]—the set of first *p* positive numbers, with $\sigma (i)=\sigma_{i}$. A BN is consistent with an ordering $\left\{v_{\sigma_{i}}\right\}$ if the parents of $v_{\sigma_{i}}$ are a subset of $\{v_{\sigma_{j}}\},j<i$, i.e. $G_{\sigma_{i}}\subseteq \{v_{\sigma_{j}}\},j<i$.

In the score-based exact approaches for optimal BN structure learning, a scoring function is used. The scoring function gives a real value (score) representing the goodness of fit of a BN to the data. To find a best BN, this score is maximized over all the possible BNs. There are a variety of scoring functions available—for example, BIC/MDL [29–31], BDeu [32, 33], and BGe [33–35]. In Causnet and CausNet-partial, we provide BIC (Bayesian information criterion) and BGe (Bayesian Gaussian equivalent) scoring function options. These two scoring functions have been shown to perform consistently well for large data, and for both discrete and continuous variables [31, 32].

## 3 CausNet

CausNet [21] uses the dynamic programming approach to finding a best BN, following the work by Koivisto & Sood [11, 12], Silander and Myllymäki [13] and by Singh and Andrew [14]. CausNet follows the algorithm proposed by Silander and Myllymäki [13] (SM algorithm henceforth) and makes heuristic modifications to it to prune the search space. It uses ‘generational orderings’ based search with parent set identification and resulting parent set constraints, as well as in-degree constraints for this purpose.

The SM method and CausNet use an an important fact about DAGs that every DAG contains at least one sink (a node with no outgoing edges). The problem of finding a best BN given the data 𝒟 relies on finding a best sink for the whole set of nodes. This in turn requires finding a best sink for the set of nodes without that sink, leading to the following recursion :

bestScore(V)=bestscore(V⧵{s})+bestScore(s,V⧵{s}), (1)

where *s* is the best sink, and *bestscore*(*V*) is the score of a best network with nodes *V*, and *bestScore*(*s*,*U*) is the best score of *s* with parents in *U*. The result is an ordering of the nodes, with each best sink removed progressively, $\left\{v_{\sigma_{i}}\},i\in \{1,2,\ldots ,p\right\}$, and their best parents from which the best BN can be recovered.

To implement the above recursion, we use a local score for a node $v_{i}$ with parents $parents(v_{i})$ using a scoring function (BIC or BGe). Then the score of a network network *G* is defined as :

$$score(G)=\sum_{i=1}^{p}localscore(v_{i},G_{i}),$$ (2)

where *localscore*(*x*,*y*) gives the score of *x* with parents *y* in the network *G*. The best score for each node $v_{i}$ with possible parents *pp*_*i*_ is then computed and the best parents *bps*_*i*_ found for $v_{i}$ :

$$bestScore\left(v_{i},pp_{i}\right)=\max_{g\subseteq pp_{i}}localscore(v_{i},g),$$ (3)

$$bps_{i}(pp_{i})=\underset{g\subseteq pp_{i}}{\text{argmax}}localscore(v_{i},g).$$ (4)

Then the best sink *s* and the best score for a best network in *V* can be found by the following equations.

$$bestSink\left(V\right)=\underset{s\in V}{\text{argmax}}bestscore(V⧵\left\{s\right\})+bestScore(s,V⧵\left\{s\right\}).$$ (5)

$$bestscore(V)=\max_{s\in V}bestscore(V⧵\left\{s\right\})+bestScore(s,V⧵\left\{s\right\}).$$ (6)

**Fig 1:**
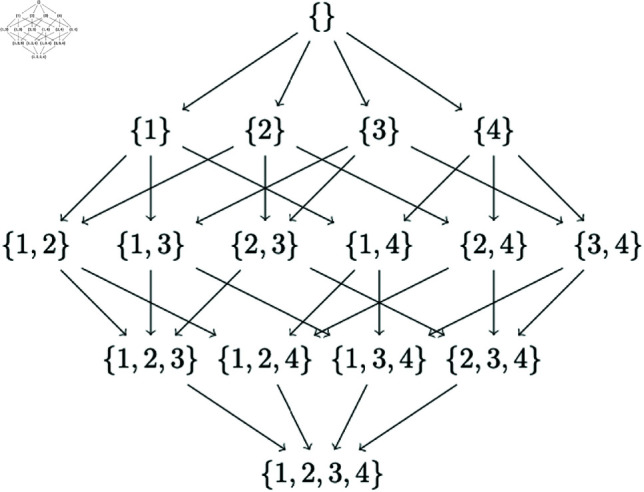
Subset lattice for BNs with four nodes {1,2,3,4}.

In Fig 1, we show the subset lattice of four nodes {1,2,3,4}; all the paths in the lattice denote node orderings that need to be searched to find a best network. Each arrow in the lattice also encodes a sink—if *p*_*s*_ is the subset at the source and *p*_*h*_ the subset at the end of a directed edge, the sink is given by $p_{h}⧵p_{s}$. In the exhaustive search, there are 4! paths that need to be searched if we know the best parents for each node in the ordering (which is computed beforehand in the DP approach). The basic CausNet approach without restricting the search space, has the following five steps:

1. Compute local scores for all $\text{p}2^{\text{p}-1}$ (node, node set)-pairs.
2. Find best parents of each of the $\text{p}2^{\text{p}-1}$ (node, possible parent set)-pairs using local scores.
3. Find a best sink for each of the $2^{\text{p}}$ node sets.
4. Using the results from Step 3, find a best ordering of the nodes.
5. Find a best network using results of Steps 2 and 4.

The CausNet algorithm, however, doesn’t search the whole space of possible BNs but searches a much smaller subspace based on parent set identification and searches only the paths that are consistent with ‘generational orderings’ resulting from the parent set constraints. Here we reproduce the algorithms of the CausNet method. For details, the reader is referred to [21].

**Algorithm 1.** Find pp, Compute feasSet and feasSetData.

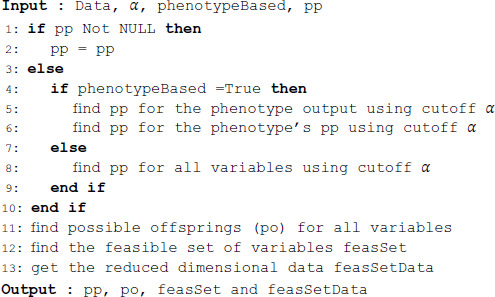



In Algorithm 1, we find the possible parents *pp*_*i*_, and also, as a result, the possible offsprings *po*_*i*_ for each node $v_{i}$ using the magnitude of a marginal association effect estimate or a False Discovery Rate (FDR) cutoff α. This step may result in a much smaller subset of nodes *feasSet* to be considered. If the response is a survival outcome, we evaluate associations using Cox proportional hazards regression, independently for each feature. In the CausNet software, we also provide an option for a ‘phenotype driven search’ for possible parents in which we consider only two or three levels of associations (similar to 1-hop and 2-hop approaches respectively in [36, 37]) starting with the outcome variable, i.e. we identify possible parents, ‘grandparents’ and ‘great-grandparents’ of the phenotype outcome.

**Algorithm 2.** Compute local scores for feasSet nodes.

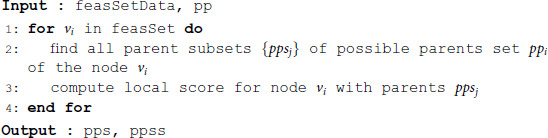



**Algorithm 3.** Compute best scores and best parents for feasSet nodes in all parent subsets.

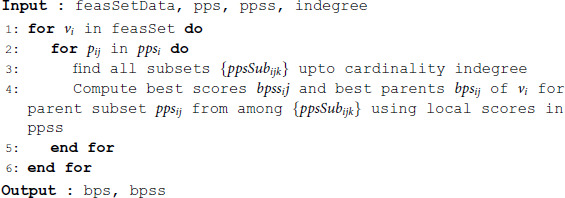



**Algorithm 4.** Compute best sinks for feasSet subsets.

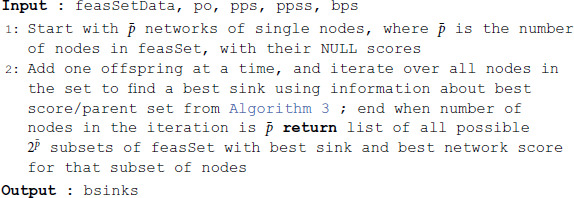



**Algorithm 5.** Find the best network(s).

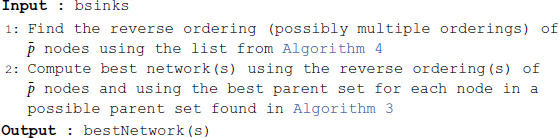



## 4 CausNet-partial—Dynamic programming on the space of ‘partial generational orderings’

In Causnet-partial, we introduce the space of ‘partial generational orderings’ of nodes instead of the full generational orderings to find small and sparse optimal BNs. This space of ‘partial generational orderings’ is a much smaller space and different from the space of ‘generational orderings’ which is searched by CausNet. In this approach we start not at the level of singletons in the algorithm for finding best sinks as is done in basic CausNet [21] but at a certain level of subsets of cardinality $\bar{p}-pOrd$, where *pOrd* is the length of partial orderings that we want to consider. For example, if we want to search partial orderings of length 2 for our example of 4 variables, we will start at the level of subsets of cardinality 2 instead of starting at the the empty set at the top of the subset lattice. This reduces the search space of orderings, and is useful when we are interested in smaller networks. CausNet-partial is a novel way that utilizes the working of the DP algorithm and modifies it at the stage of finding best sinks. It considers smaller orderings of nodes instead of full orderings of nodes. Furthermore, these ‘partial generational orderings’ are consistent with the parent set constraints.

The bestSinks algorithm of basic CausNet (Algorithm 4) is modified to Algorithm 6 in case of CausNet-partial. Here, we start at the level of subsets of cardinality $\bar{p}-pOrd$. At this level, each element in a subset is considered as a sink with the rest of elements as possible parents. The best sink is computed to be the one that gives the best score with parents among the rest of the elements. Following this, the rest of the bestSinks algorithm proceeds as in the case of the original CausNet.

**Fig 2:**
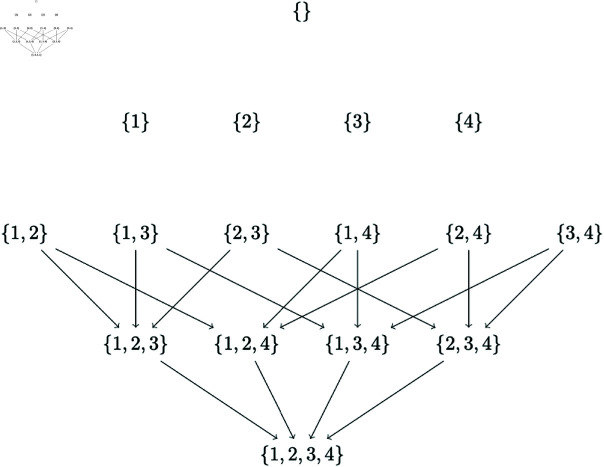
Subset lattice for partial orderings with pOrd=2 on four nodes {1,2,3,4}.

The subset lattice to be explored for partial orderings of length 2 for our example of 4 variables reduces to the one in Fig 2. Observe that in this case, without parent set constraints, we are searching only 2×(42)=12 orderings, instead of 4! orderings as is the case with original CausNet without parent set constraints.

**Fig 3:**
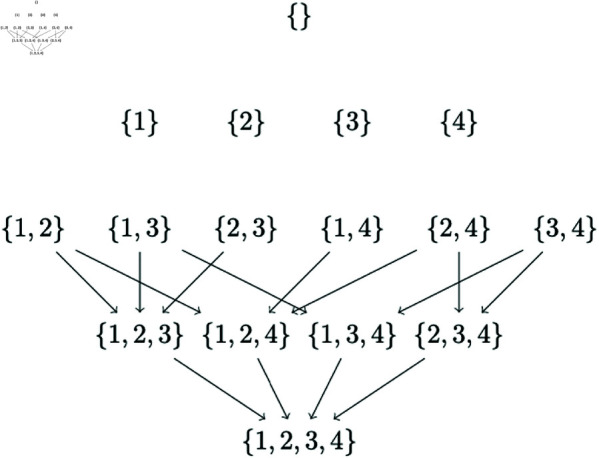
Subset lattices with parent set constraints. The parent set constraints are given by : *pp*_1_ = {2,4}, *pp*_2_ = {3,1}, *pp*_3_ = {2}, *pp*_4_ = {1}.

Now suppose that the possible parent sets for the four nodes {1,2,3,4} are as follows : *pp*_1_ = {2,4}, *pp*_2_ = {1,3}, *pp*_3_ = {2}, *pp*_4_ = {1}. Factoring in these possible parent sets, we get the subset lattice as in Fig 3. Observe that the partial orderings of 2 nodes to be searched has further reduced to only 9. These 9 partial orderings are what we call ‘partial generational orderings’. Every node in a ‘partial generational ordering’ has a possible parent in the preceding set of nodes as per the possible parent set constraints. Observe that the missing arrows in Fig 3 compared with Fig 2 are the result of the parent set restrictions. For example, there is no arrow from the subset {2,3} to {2,3,4} because the node {4} as a sink has no parent in the set {2,3}- the only possible parent of node {4} is {1}. Observe that the total number of orderings to be searched has reduced from 24 (in CausNet) to 9 (in CausNet-partial).

**Fig 4:**
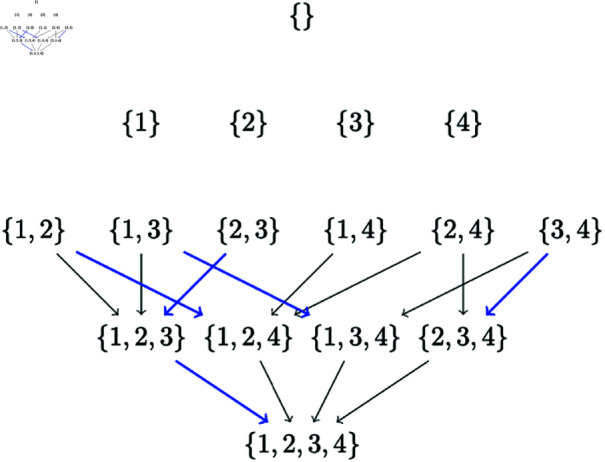
Subset lattices with parent set constraints and best sinks identified by CausNet-partial as an example. The blue arrows encode the best sink for each subset in the lattice. As an example, the blue arrow from {3,4} to {2,3,4} encodes the best sink {2}. The complete blue path represents the best network.

Now suppose the best sinks are given by the ones encoded by blue arrows as in Fig 4. Then the best BN is given by the reverse ordering (4,1) encoded by the path given by the complete path of blue arrows from top to bottom. The first blue arrow in this path encodes the best sink {1} in the set {1,2,3}, and the second blue arrow encodes the best sink {4} in the set {1,2,3,4}.

Search for sparse networks in the space of Partial Orderings reduces the run time of the best sinks algorithm substantially, and thus of the CausNet-partial method.

**Algorithm 6.** Compute best sinks for feasSet subsets—partial orderings.

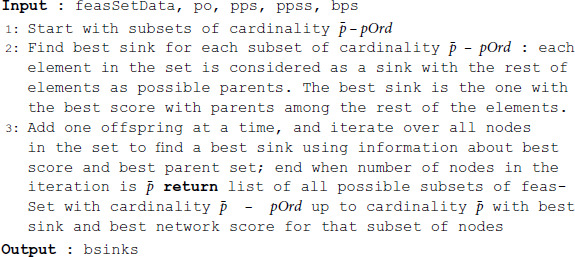



**Theorem 4.1.** *Total number of labeled DAGs with p nodes and partial order r is* 𝒪(p!(p−r)!2(p2)2(p−r2)).

*Proof:* For *p* nodes, total number of orderings is p!; With *pOrd* = *r*, the number of orderings with *r* nodes is $\frac{p!}{\left(p-r\right)!}$. For each node in the partial ordering of *r* nodes, the number of possible parent sets is given by-


&2(p−1)2(p−2)…2(p−r)&=2(p−1)+(p−2)…+(p−r)&=2(∑1(p−1)k−∑1(p−r−1)k)&=2(p2)2(p−r2).

So, the total number of labeled DAGs with *p* nodes and partial order *r* is given by :


p!(p−r)!2(p2)2(p−r2).

The number of network structures is 𝒪(p!(p−r)!2(p2)2(p−r2)) because there are many repeated structures in this combinatorial computation; e.g. there are r! structures with all *r* nodes disconnected. ◻

**Theorem 4.2.** *Without parent set and in-degree restrictions, the CausNet-partial -the partial generational ordering based DP algorithm—explores all the* 𝒪(p!(p−r)!2(p2)2(p−r2)) *network structures for p nodes*.

*Proof:* Without parent set restrictions, every node is a possible parent of every other node. In Fig 1, let k,0≤k≤p be the cardinality of subsets in the subset lattice for *p* nodes. Let each row in the subset lattice be the *k*th level in the lattice. Now adding a new element in Algorithm 4 corresponds to an edge between a subset of cardinality *k* and *k*–1, which considers the added element as a sink in the subset of cardinality *k*. Number of edges to a subset of cardinality *k* from subsets of cardinality *k*–1 is given by(kk−1). The number of possible parent combinations for a sink in subset of cardinality *k*, without in-degree restrictions, is given by $2^{\text{k}-1}$. In Algorithm 3, we explore all these possible parent sets to find the best parents for each sink *s* in each subset at each level *k*. The Algorithm 6 uses this information to get the best sink (possibly multiple) for each subset at level *k*. Thus the total number of networks searched by the Causnet-partial algorithm is given by


∏k=(p−r)p(kk−1)2k=(p−rp−r−1)(p−r+1p−r)(p−r+2p−r+1)…(p−1p−2)(pp−1)2p−r2p−r+1…2p−1=p!(p−r)!2(p2)2(p−r2).

◻

## 5 Simulations

We compare CausNet and CausNet-partial with three state-of-the-art methods that are currently extensively used for optimal BN structure learning. One is an exact method called GOBNILP [15, 16], which is an integer learning based method, while the other two—BNlearn’s Hill Climbing (HC) [38] and Max-min Hill Climbing (MMHC) [39]—are approximate methods. While Hill-Climbing (HC) is a score-based method that uses greedy search on the space of the BNs, the Max-Min Hill-Climbing (MMHC) is a hybrid method that uses a constraint-based algorithm, Max-Min Parents and Children (MMPC), to find skeleton of optimal undirected graph, followed by a score-based search to find directions of the edges.

Bayesian networks were simulated as in [21] by generating *N* x *p* data matrices of continuous Gaussian data with the dependencies between variables simulated using linear regression with varying effect sizes. For details of simulation design, the reader is referred to [21]. Again as in [21], we use the False Discovery Rate (FDR) and Hamming Distance as the metrics to compare the methods. With *FP* being the number of false positives and *TP* the number of true positives,


$$FDR=\frac{FP}{FP+TP}.$$

FDR is a especially useful metric for high dimensional data and for network analysis [40]. Reducing FDR is an important indicator of the quality of BN given the data, where we want to identify as many true edges while simultaneously reducing false positive edges.

The Hamming distance is a measure of structural similarity of BNs, and gives a measure of goodness of a predicted BN [41, 42]. With *FP* representing the number of false positives and *FN* the number of false negatives, Hamming Distance is defined as :


HammingDistance=FP+FN.

In the first set of simulations, we ran the methods on simulated BNs with number of variables below 100, i.e. p=10,20,40,50,60,100, and number of data samples N=500,1000,2000. For using CausNet and CausNet-partial, we used FDR cutoff of 0.3 for parent set identification and an in-degree of 2 for all the experiments. In [21], we have shown how CausNet compares with the three other methods. Here we include CausNet-partial in the two Tables 1 and 2. We use *pOrd* = 3 for search on partial orderings in CausNet-partial. In these tables, we show the average *FDR* for different methods in finding both directed and undirected graphs. The results for CausNet and CausNet-partial with the BIC scoring function are given. The results show that our methods—both CausNet and CausNet-partial—outperform the other three methods for the most part.

**Table 1:** FDR (p=10,20,40,N=500,1000,2000).

Method	FDR(undirected)	FDR(directed)
CausNet	0.229	0.412
CausNet-partial	0.22	0.32
Gobnilp	0.312	0.345
BN-HC	0.466	0.577
BN-MMHC	0.368	0.511

**Table 2:** FDR (p=50,60,100,N=500,1000,2000).

Method	FDR(undirected)	FDR(directed)
CausNet	0.359	0.494
CausNet-partial	0.22	0.32
Gobnilp	0.540	0.560
BN-HC	0.635	0.694
BN-MMHC	0.787	0.812

For the number of variables up to 40 (Table 1), on the metric of FDR, CausNet-partial performs the best, while Gobnilp is the second best and Causnet the third best for directed graphs. For undirected graphs, CausNet-partial and CausNet are the first and second best performers followed by Gobnilp. For the number of variables between 50 and 100 (Table 2), our methods are the top two best for both directed and undirected graphs.

**Fig 5:**
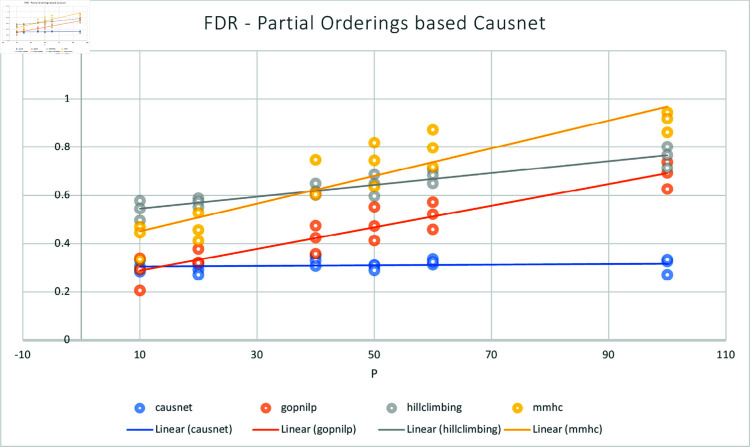
Average FDR with p=10,20,40,50,60,80,100 and N=500,1000,2000. Here the CausNet uses Partial orderings based search and uses parent set identification for dimensionality reduction while other algorithms don’t use any parent set identification.

**Fig 6:**
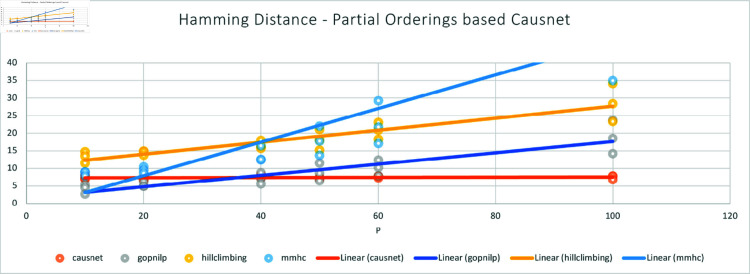
Average Hamming Distance with p=10,20,40,50,60,80,100 and N=500,1000,2000. Here the CausNet uses Partial orderings based search and uses parent set identification for dimensionality reduction while other algorithms don’t use any parent set identification.

The Figs 5 and 6 show the comparison of CausNet-partial with the three methods on the metrics of FDR and Hamming distance. The FDR for Partial Orderings based Causnet (CausNet-partial) is the best for number of variables greater than 10; Gobnilp is the best for 10 variables and the second best for number of variables greater than 10. In terms of Hamming distance, CausNet-partial again is the best for variables more than 20; Gobnilp and MMHC have lower Hamming distance than that of Causnet for variables less than 20, but rise quickly for variables more than 40; overall, Gobnilp is again the second best.

### 5.1 Number of variables upto 1000

**Fig 7:**
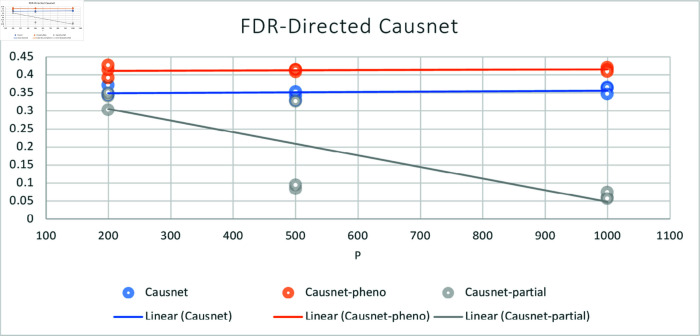
Average FDR with p=200,500,1000 and N=500,1000,2000. Here we compare the three versions of Causnet—Causnet basic, Phenotype based Causnet and Partial Orderings Causnet.

**Fig 8:**
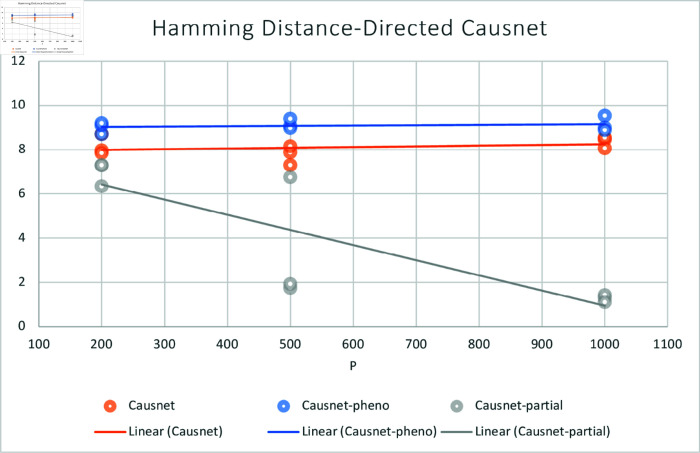
Average Hamming Distance with p=200,500,1000 and N=500,1000,2000. Here we compare the three versions of Causnet—Causnet basic, Phenotype based Causnet and Partial Orderings Causnet.

For these simulations, we use number of variables p=200,500,1000, and number of data samples N=500,1000,2000. For these simulations, we don’t compare with the other three algorithms as they either can not handle such high number of variables or take many orders of magnitude longer to process. Instead, we compare the three variants of CausNet—CausNet [21], phenotype-driven CausNet [21], and CausNet-partial. The results are shown in Figs 7 and 8. Here we observe that Partial Orderings Causnet gives the best results for both FDR and Hamming distance. Furthermore, both the FDR and Hamming Distance for Partial Orderings Causnet keeps getting better as the number of variables increase. This can be explained by the fact that as we reduce the length of orderings being searched there is a lesser chance of false positives. This also shows the effectiveness of using partial orderings where we expect sparse networks while the original data has much higher number of features, a common scenario for high dimensional data.

### 5.2 Runtime

Search for sparse and small networks in the space of Partial Orderings reduces the run time of the best sinks algorithm substantially, and thus of the Causnet-partial method. To show the runtime gains expected from theory as shown in Sect 4, we compare the runtimes of the five algorithms. During the simulations, it was observed that Gobnilp mostly terminates without producing any output BN for *p*>100. Due to this reason, we split the comparison for p≤100 and 100<p≤1000. The average runtimes are shown in Table 3. Causnet-partial and Causnet have the top best runtimes for p≤100. For 100<p≤1000, Causnet-partial and CausNet are better than Gobnilp and MMHC. Although HC is faster for more than 100 variables, we have shown in the sections above that its accuracy is not so good when compared with Causnet-partial and CausNet. Between Causnet-partial and CausNet, Causnet-partial gives substantially reduced runtime, further validating the utility of Causnet-partial for finding sparse small optimal BNs.

**Table 3:** Average Runtime in seconds.

	p≤100	100<p≤1000
CausNet	0.025	277.87
CausNet-partial (pOrd 3)	0.018	123.7
Gobnilp	99.8	*
HC	0.088	68.17
MMHC	0.288	18763.493

*Terminates without output.

## 6 Application to benchmark Bayesian network—ALARM

Having shown the superior performance of CausNet and CausNet-partial, we show the efficacy of our method in finding sparse BNs now based on partial orderings. To this end, we apply our method to a benchmark Bayesian network ALARM [43]. The ALARM (“A Logical Alarm Reduction Mechanism") is a Bayesian network designed to provide an alarm message system for patient monitoring. The data set contains 37 variables, all discrete, with number of categories between 2 and 4. The dataset was downloaded from the bnlearn website (https://www.bnlearn.com/bnrepository/). We used bootstrap samples of size 1000, taken from the alarm data set, which has a total sample size of 20,000.

**Fig 9:**
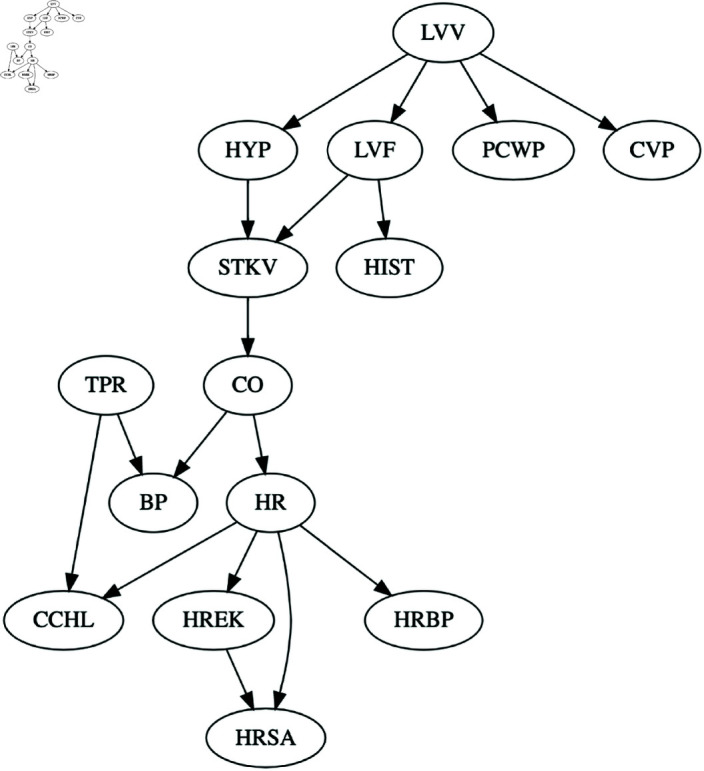
Optimal ALARM BN discovered by CausNet.

**Fig 10:**
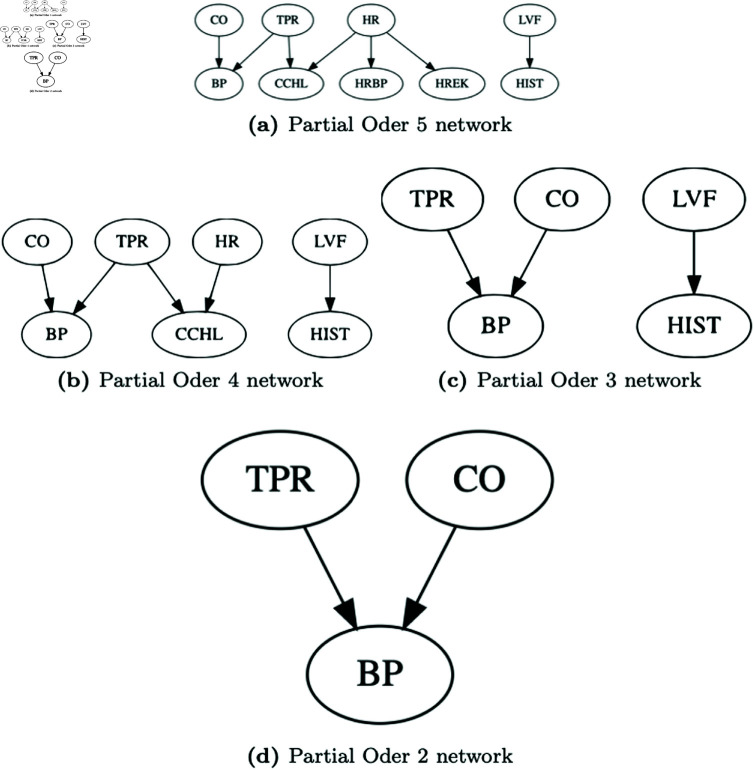
ALARM networks with different partial orderings.

We first apply the original CausNet with discrete option for BIC score. Then we apply CausNet-partial varying the partial order from 5 to 2. Fig 9 shows the BN discovered by CausNet, and Fig 10 shows the BNs discovered by CausNet-partial with different partial orders. CausNet discovers a BN with 15 nodes when using the in-degree 2 and the FDR cut-off of 0.3 for possible parent set identification. With the same in-degree and the FDR cut-off, we then apply the CausNet-partial to obtain sparser BNs based on partial generational orderings. The results are shown in Table 4. As we can see from the figures and the table, we get smaller and smaller networks as we reduces *pOrd*. The FDR for CausNet-partial is a bit higher than the one we get with CausNet, but this keeps getting better as pOrd is reduced. FDR for undirected networks is consistently 0 for CausNet-partial. Initial rise in FDR is due to substantial reduction of nodes from CausNet to CausNet-partial. The nodes and the directed edges in the partial order 5 network are subsets of nodes and the directed edges in the CausNet network, as expected. The lower partial order networks maintain the same consistency with the directed edges. The gain in speed of computation is illustrated well with the runtime decreasing by many orders of magnitude going from CausNet to CausNet-partial.

**Table 4:** FDR and Runtime for ALARM.

	No. of nodes	FDR (directed)	FDR (undirected)	Runtime
CausNet	15	0.35	0.05	153
CausNet-partial (pOrd 5)	9	0.55	0	41.6
CausNet-partial (pOrd 4)	7	0.42	0	21.9
CausNet-partial (pOrd 3)	5	0.2	0	16.6
CausNet-partial (pOrd 2)	3	0	0	15.9

## 7 Application to clinical trial data

To demonstrate the effectiveness of CausNet-partial in the case of a survival outcome and continuous data, we applied our method to a recently published data on ovarian cancer. This dataset [44] includes gene expression data of 513 genes collected from participants in multiple clinical trials. This study aimed to develop a prognostic signature based on gene expression for overall survival (OS) in patients with high-grade serous ovarian cancer (HGSOC). Expression of 513 genes, selected from a meta-analysis of 1455 tumors was measured using NanoString technology from formalin-fixed paraffin-embedded tumor tissue collected from 3769 women with HGSOC. Results of this study showed that expression levels of 276 genes were associated with OS (false discovery rate <0.05) in covariate-adjusted single-gene analyses.

**Fig 11:**
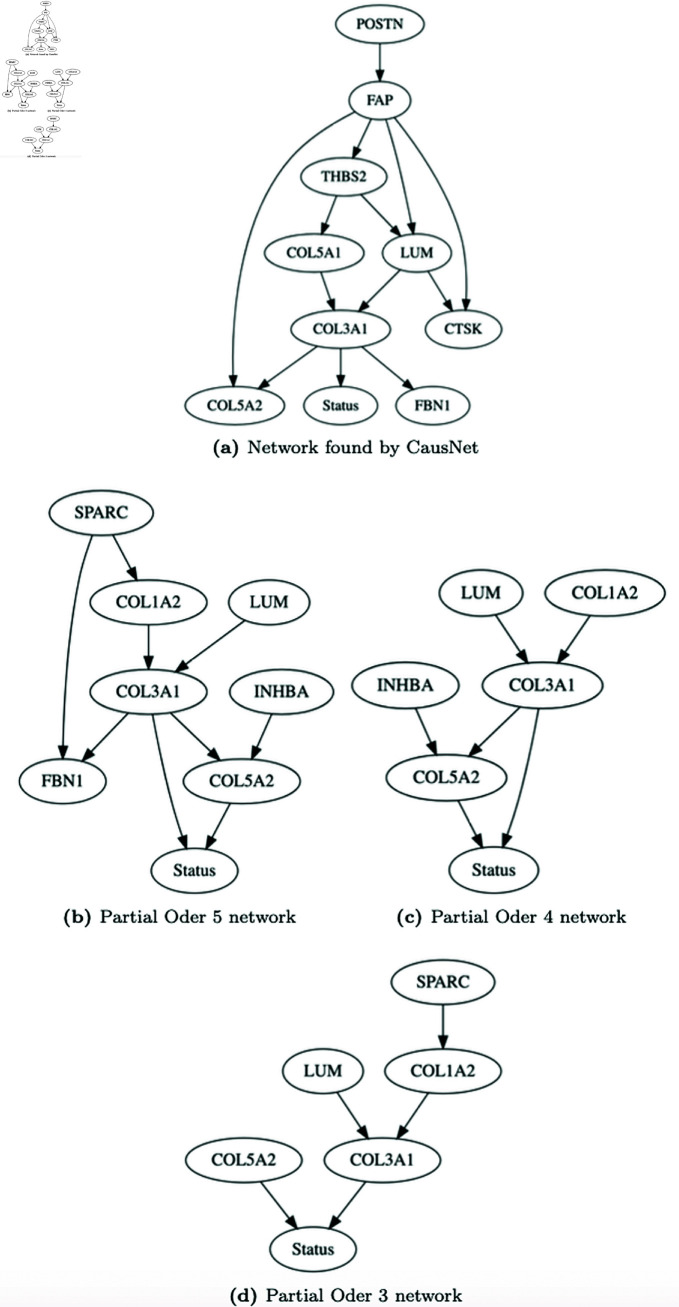
Optimal ovarian cancer BNs learned by CausNet and CausNet-partial.

We first reduce the dimensionality of data by finding parent sets using FDR and correlation cut-offs. For this, we used a three-level phenotype driven search. For the disease node ‘Status’, dead or alive at censoring or end of follow-up, we carried out Cox proportional hazard regression for each gene separately, adjusted for age, stage, and stratified site. Then we computed analysis of variance (ANOVA) tables for the fitted models and created a list of *p*-values based on the $\chi^{2}$ distribution. FDR was then computed using the Benjamini & Hochberg (BH) method. We choose the 5 genes, *ZFHX4*, *TIMP3*, *COL5A2*, *FBN1*, and *COL3A1*, with the most significant p-values as possible parents of the disease node. For rest of the nodes, we use correlation cut-offs to identify possible parent sets of each of the other nodes. Using the indegree of 2 and tuning the correlation cut-off parameter, we arrive at a ‘feasSet’ of 16 genes with non-null parent sets. We then apply first the CausNet and then CausNet-partial, varying the partial orders from 5 to 3. The resulting optimal BNs learned are shown in Fig 11. On a personal computer with a 2.3 GHz Intel Core i9 processor with 16 GB RAM, the processing for each network took less than 5 minutes. As expected, there are substantial overlap of genes and the edges in the BNs discovered by CausNet and CausNet-partial. But there are also differences, with some different genes and some different edges in the networks. Some differences are explained by the fact that there are multiple best BNs for each of the cases, and we pick the first one given by the system. These differences can be further analyzed over multiple best BNs for statistical uncertainty quantification.

In a recent work [45] on finding ovarian cancer signature, Ye et, al. found that the tumors highly express a panel of genes, namely POSTN, LUM, THBS2, COL3A1, COL5A1, COL5A2, FAP1 and FBN1. All these eight genes are in the BN found by CausNet, while partial order smaller BNs have a subset of these genes as expected. Observe that FAP and FAP1 in [45] are the same gene (while the abstract mentions FAP, Table 1 mentions it as FAP1). The role of the pool of genes in the BNs learned by CausNet and CausNet-partial is further supported by recent research in [46] (FBN1) and [47] (COL5A1). This further shows the efficacy of CausNet and CausNet-partial in learning small and sparse networks leading to disease outcome. The networks produced by CausNet-partial can be used for further experimental design and validation.

## 8 Discussion

In this work, we implemented an extension to our earlier method CausNet. This extension—CausNet-partial—is a novel, simple and highly efficient method to find optimal small and sparse BNs using dynamic programming with parent set identification and constraints. Our main novel contribution is the revision of the SM algorithm [13] to identify possible parent sets, reduce the search space with parent set constraints and in using ‘partial generational orderings’ based search. This search method is a more efficient and cost-effective way to explore the search space for small and sparse BNs than the original approach which is based on lexicographical ordering. This enables our method to be applied to large dimensional data effectively, while the SM algorithm can not be applied to more than 30 variables [13].

In simulations, our algorithm outperforms the three state-of-the-art algorithms that are widely used currently for optimal BN discovery. We further show its application to a benchmark discrete Bayesian network ALARM, a Bayesian network designed to provide an alarm message system for patient monitoring. We first apply the original CausNet and then CausNet-partial, varying the partial order from 5 to 2. CausNet-partial discovers small sparse networks with drastically reduced runtime as expected from theory developed as part of this work.

We also showed a usecase application of CausNet-partial to a recently published data involving gene expression of 513 genes and ovarian cancer prognosis with a survival outcome. The BNs learned by CausNet and CausNet-partial included most of the genes that have been shown to be associated with ovarian cancer as reported in [45–47]. While the BN learned by CausNet included all the genes reported in these papers, the smaller BNs learned by CausNet-partial had a subset of these genes as expected. As most existing methods, including the ones in our study, do not provide support for survival outcomes for BN structure learning, our method fills an important gap in providing a method that works for large data with survival outcome.

In conclusion, our partial generational orderings based search for small optimal BNs, CausNet-partial, is an efficient and highly scalable approach for finding optimal sparse and small Bayesian Networks and can be applied to high dimensional data. Using specifiable parameters—correlation, FDR cutoffs, in-degree, and partial order—one can increase or decrease the number of nodes and density of the networks, as required by the application domain.
